# Response to “The action of Arabidopsis DICER-LIKE2 in plant growth inhibition”

**DOI:** 10.1093/plcell/koaf163

**Published:** 2025-08-29

**Authors:** Carsten Poul Skou Nielsen, Laura Arribas-Hernández, Peter Brodersen

**Affiliations:** Novo Nordisk A/S, CMC API Development, Hagedornsvej 1, Gentofte 2820, Denmark; Instituto de Hortofruticultura Subtropical y Mediterránea La Mayora (IHSM), Universidad de Málaga—Consejo Superior de Investigaciones Científicas (UMA-CSIC), Boulevard Louis Pasteur 49, málaga 29010, Spain; Department of Biology, University of Copenhagen, Ole Maaløes Vej 5, Copenhagen DK-2200, Denmark

In their letter entitled “The action of Arabidopsis DICER-LIKE2 in plant growth inhibition”, authors from the Hongwei Guo laboratory (Liu et al. 2025) bring up several points to question the validity of our evidence for a new understanding of the function of the DICER-LIKE ribonuclease DCL2. Two broad matters are brought up. First, our evidence for an RNA interference (RNAi)-independent role of DICER-LIKE2 (DCL2) in conferring growth restriction in mutant backgrounds lacking the major cytoplasmic double-stranded RNA-directed ribonuclease DCL4, described in a paper published in *The Plant Cell* last year (Nielsen et al. 2024). Second, our evidence that this growth restriction is in fact a manifestation of DCL2-mediated sensing of double-stranded RNA that switches on innate immunity dependent on specific nucleotide-binding leucine-rich repeat receptors (NLRs), described in a preprint available to the community (Nielsen et al. 2023).

We welcome the initiative by Liu et al. to further debate the evidence for these RNAi-independent roles of DCL2 and thank the editors for allocating space in the pages of *The Plant Cell* for this debate.

We assume that readers of this response will have read the letter of Liu et al. and, therefore, we do not introduce basic notions on RNAi and DCL ribonucleases again. Nonetheless, we remind the reader of three points, key to the debate that follows:

1. RNAi is understood to be the most important element of basal antiviral immunity in plants (Ding and Voinnet 2007).
2. Antiviral RNAi is understood to involve two redundantly acting DCL enzymes: DCL4 and DCL2 (Ding and Voinnet 2007).
3. Often, DCL2 is described as a back-up of DCL4, because in the vast majority of cases, DCL2-produced siRNAs only become detectable when DCL4 is inactivated, either genetically or through viral anti-RNAi virulence factors (Deleris et al. 2006; Wang et al. 2011; Andika et al. 2015).

In addition, we hope the reader will forgive us for taking them on a slight detour whose purpose is to provide the necessary background to understand our direct answers to the points raised by Liu et al. This detour involves three elements:

1. The basic observations at the core of the debate.
2. How they have been explained prior to our contributions.
3. What the main problem with these explanations is.

## Point 1. Basic observation

In the absence of DCL4, Arabidopsis plants display a growth phenotype with incomplete penetrance (Gasciolli et al. 2005; Parent et al. 2015; Wu et al. 2017). This phenotype is exacerbated by partial loss of DCL1 function (Bouche et al. 2006), or, as shown by the Guo laboratory, by inactivation of RNA decay factors (Zhang et al. 2015), or, as we show in our papers, by inactivation of the HSP90 cochaperone SGT1B (Nielsen et al. 2023; Nielsen et al. 2024). In these genetic backgrounds, the penetrance is 100% and the phenotype is more severe than in *dcl4* single mutants. In all cases, the growth phenotypes can be completely suppressed by inactivation of the *DCL2* gene (Bouche et al. 2006; Zhang et al. 2015; Nielsen et al. 2023; Nielsen et al. 2024).

## Point 2. Explanation prior to our contributions

The Guo laboratory and others have advocated the following explanation for the ability of DCL2 to confer growth phenotypes in the absence of DCL4: DCL2 makes ectopic siRNAs, 22 nucleotides (nt) in size. Others have shown that the 22-nt size of small RNA is important because in contrast to 21-nt siRNAs, 22-nt siRNAs efficiently trigger siRNA amplification through the RNA-dependent RNA polymerase, RDR6 (Chen et al. 2010; Cuperus et al. 2010; Yoshikawa et al. 2013; Fei et al. 2018; Sakurai et al. 2021; Yoshikawa et al. 2021). The ectopic siRNAs resulting from DCL2 activity and uncontrolled amplification, particularly those targeting the mRNAs *SMXL4/5—*encoding chaperonin-like factors—and *NIA1/2—*encoding nitrate reductases—are then proposed to cause growth phenotypes via silencing through the RNAi pathway (Zhang et al. 2015; Wu et al. 2017, 2020). This explanation is pleasing at first sight and clearly consistent with established knowledge. After all, siRNAs can silence gene expression through RNAi, and the genes in question are related to growth and metabolic activity.

## Point 3. Main problem with the explanation prior to our contributions

There is one major problem with the explanation described above, however: there is no proof for it. Clearly, the siRNA populations in question coincide with the growth phenotype, but so do many other transcriptomic changes that are not suggested to cause the phenotypes. Why this difference in treating similar evidence? Simple guesswork? Not quite, but almost. The authors rely on the fact that double knockout mutations in the genes concerned can produce phenotypes “similar” to those obtained in *dcl4* mutants. Unfortunately, this is not a strong argument. First, it assumes comparable molecular effects obtained by genetic knockouts and ectopic siRNA populations, but in contrast to the knockouts, it is unknown when, where, and to what extent the siRNAs silence the targets in question. Second, it clearly does not prove that the siRNAs cause the phenotype, because such proof of causality would require the specific reversal of the gene silencing proposed to underlie the phenotype.

There is one experiment used by the RNAi field for more than two decades to resolve this crucial question of causality: engineer a target resistant to siRNA/miRNA-guided silencing and test whether expression of this target is sufficient to suppress the phenotypes observed. As explained in our paper (Nielsen et al. 2024), this is not straightforward to achieve experimentally for at least two reasons. First, the implication of an entire siRNA population, not just a single small RNA species, necessitates extensive re-engineering of the target in question. Second, we do not know exactly which properties of the *NIA* and *SMXL* genes cause RNAi initiation, and if engineered versions retain those properties, the experiment would be compromised. Thus, the experiment may be cumbersome, although not outright impossible, to carry out. Unfortunately, results of such an experiment have not been reported thus far.

## An alternative explanation for growth defects conferred by DCL2 in the absence of DCL4

Our work provides evidence for a completely different model of DCL2 function that explains the growth restriction phenotype of *dcl4* (and other RNA metabolism mutants) as a manifestation of autoimmunity. In this model, DCL4 and DCL2 both perform crucial, yet different functions in basal antiviral immunity. DCL4 mediates classical RNAi, but DCL2 provides a second layer of immunity that only comes into play when RNAi has been defeated, and cytoplasmic double-stranded RNA (dsRNA) concentrations are abnormally high. In that situation, the very processing of dsRNA by DCL2 is a signal that switches on intracellular NLRs to activate an innate immune state that includes growth restriction. Hence, in *dcl4* mutants, DCL2 processes endogenous dsRNA, switching on NLR-mediated immunity that explains the DCL2-dependent growth phenotypes. Clearly, this model predicts:

- that DCL2 causes growth restriction independently of a silencing function of the siRNAs that it produces,
- that activation of NLRs actually causes the growth restriction seen in *dcl4* mutants,
- that those NLRs have a role comparable to that of DCL2 in basal resistance to at least one virus rendered incapable of RNAi suppression.

The letter by Liu et al. raises critical points on selected pieces of the evidence in support of this model but omits other crucial points of evidence from our papers. Below, we provide answers to what we consider to be the five main points raised. Wherever relevant, we also include mention of evidence from our studies omitted in the letter by Liu et al.

## Five main points of criticism by Liu et al.

### Point 1. Small RNA-sequencing depth

**Figure 1.**
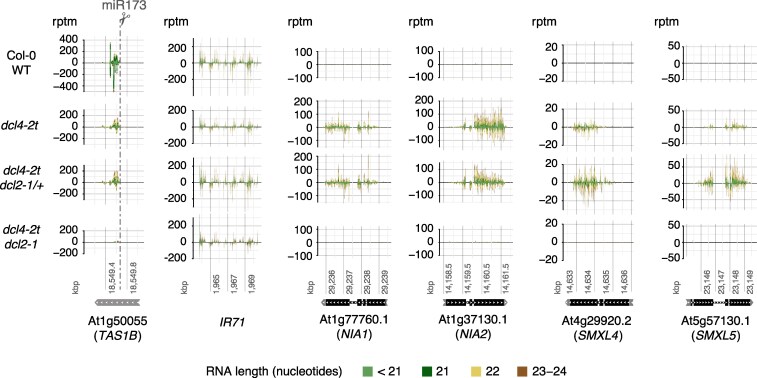
Full DCL2 dosage is not required for siRNA production. Analyses of small RNA-Seq data from 21-day-old plants of the indicated genotypes, mapped onto *TAS1B*, *INVERTED REPEAT 71* (*IR71*), *NIA1*, *NIA2*, *SMXL4*, and *SMXL5*. rptm, reads per ten million. The results shown in this figure were first published as elements of Figure 4 in our 2024 Plant Cell paper (Nielsen et al. 2024).

Liu et al. point out that our small RNA-sequencing performed to get siRNA profiles of Col-0 WT, *dcl4*, *dcl4 DCL2/dcl2* (heterozygous for the *dcl2* knockout allele), and *dcl4 dcl2* has much less sequencing depth than that of other studies addressing DCL2-dependent phenotypes in *dcl4* mutant backgrounds. This is undoubtedly true, but is it relevant? The data clearly show detection of several DCL2-dependent siRNAs upon loss of DCL4, including the ones believed by Liu et al. to cause developmental phenotypes (from *NIA1/2* and *SMXL4/5* loci). The data also show that these and other DCL2-dependent siRNAs (some confirmed by small RNA blots) accumulate to comparable levels in *dcl4* and *dcl4 DCL2/dcl2* plants, and their DCL2-dependence is confirmed in the *dcl4 dcl2* double mutants (Fig. 1). Regardless of sequencing depth, the results are clear. If we were claiming differential expression of siRNAs from loci with low read counts, we would understand the concern. But in this case, there are ample reads to safely conclude that there is no decrease in siRNA levels from *NIA1/2* or *SMXL4/5* in *dcl4 DCL2/dcl2* (heterozygous for the *dcl2* knockout allele) compared to *dcl4 DCL2/DCL2* (with two wild type *DCL2* copies). This is the key point, because we show that reducing the DCL2 protein dosage by half in *dcl4 DCL2/dcl2* is sufficient to significantly reduce the penetrance of the growth restriction phenotype (Nielsen et al. 2024).

### Point 2. Experimental design: type of tissue used for small RNA profiles

Liu et al. further argue that the way we decided to collect the material for small RNA-sequencing compromises the utility of the result. This point concerns the comparison of small RNA profiles in the following mutants, whose phenotypes are noted between parentheses.

- Col-0 wild type (all plants green and healthy)
- *dcl4 DCL2* (two *DCL2* wild type alleles) (a percentage of the population shows growth restriction, chlorosis, and anthocyanin accumulation)
- *dcl4 DCL2/dcl2* (heterozygous) (a smaller percentage than in *dcl4-2* shows growth restriction, chlorosis, and anthocyanin accumulation)
- *dcl4 dcl2* (homozygous) (all plants green and healthy)

We have taken care to explain the reasoning behind the way the experiment is done in the paper (Nielsen et al. 2024). The difficulty relates to the incompletely penetrant phenotypes. Our objective was to assess whether reducing the dosage of DCL2 by half affects DCL2-dependent siRNA levels as may be expected if silencing by some of these siRNAs caused the phenotype. But if we grew the plants under conditions in which the phenotypes penetrate and selected the ones with phenotypes, we would be likely to see the same siRNAs in the same phenotypic categories of the different mutant backgrounds, simply because the generation of the siRNAs coincides with, but does not necessarily cause, the growth phenotype. In contrast, conducting the experiment the way we did, with tissue harvest prior to appearance of visible symptoms, addresses the question we wanted to address: whether reducing the dosage of DCL2 by half affects DCL2-dependent siRNA levels transcriptome-wide. The result is clear, it does not: The mRNA-derived DCL2-dependent siRNAs differentially expressed between *dcl4* and *dcl4 DCL2/dcl2* split into three categories: some unaffected, some less abundant, some more abundant, likely reflecting the transcriptomic differences that arise as a consequence of the effect of DCL2 dosage on activation of immune responses, even in presymptomatic seedlings.

We recognize that this is a choice with some imperfection. Doing the experiment in the way suggested by Liu et al. could have merit if indeed high *SMXL4/5* siRNA levels were found in nonsymptomatic *dcl4 DCL2/dcl2* heterozygous plants. We considered this possibility to be unlikely, simply because all the available evidence suggests that growth restriction coincides with (but again, is not necessarily caused by) high expression of *SMXL4/5* siRNAs. Therefore, we did not embark on yet another experiment whose results would not distinguish between causality and correlation. We stress that the results we report are useful, because they establish that reducing the DCL2 dosage by half does not appreciably affect the ability to produce steady state levels of many DCL2-dependent siRNAs compared to the full DCL2 dosage.

#### The importance of the linear dependence of phenotypic penetrance on DCL2 protein levels

**Figure 2.**
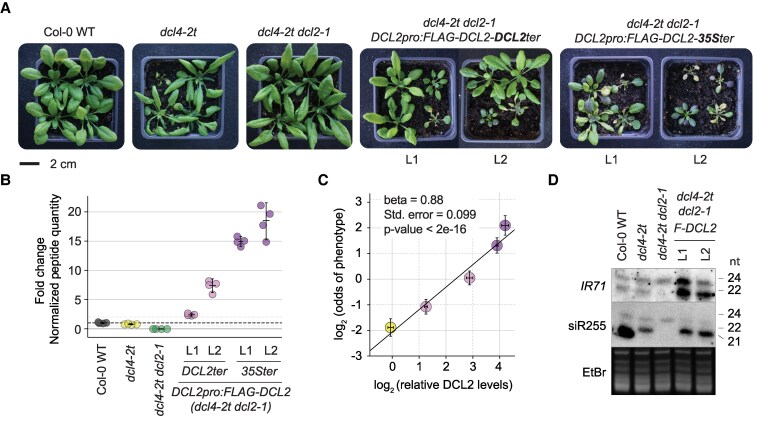
The penetrance of the DCL2-dependent phenotype is directly proportional to DCL2 protein abundance in rosette leaves. **A)** Representative individuals of 25-day-old plants of the indicated genotypes. Plants were grown in long days, first on sterile MS plates, and healthy seedlings at Day 11 were transferred to soil and grown for 14 to 15 additional days. **B)** Normalized quantities of the DCL2 peptide YVAGNNSGLQNQTR [421, 434 AA] identified by targeted mass spectrometry in the indicated independent transgenic lines, compared to Col-0 wild type plants. **C)** Dot plot showing the proportionality between the odds of having any of the phenotypes described in **(A)**, and DCL2 protein abundance (relative to endogenous DCL2 levels in *dcl4*-*2t*) as determined by targeted mass spectrometry in **(B)**. In both axes, the magnitudes are represented as log2, and error bars refer to standard error. The diagonal line represents a generalized linear mixed model with a strong linear correlation. **D)** Small RNA blot of 14-day-old sterile-grown seedlings with no apparent phenotypes, of 2 independent transgenic lines expressing *DCL2pro:FLAG-DCL2-35Ster* (*F-DCL2*) in the *dcl4-2t dcl2-1* background compared to controls. The same membrane was hybridized to radiolabeled probes complementary to endogenous siRNAs originating from *IR71* (*INVERTED REPEAT 71*), and subsequently to siR255 (a trans*-*acting siRNA) after probe stripping. Ethidium bromide (EtBr) staining of the upper part of the gel is shown as loading control. The results shown in this figure were first published as elements of Figures 2 and 5 in our 2024 Plant Cell paper (Nielsen et al. 2024).

At this point, it becomes relevant to dig a bit deeper into the dependence of growth restriction phenotypes on DCL2 protein dosage. The letter by Liu et al. touches on this, but the weight of the argument does not come through completely clearly in their letter. Hence, we summarize here what we actually report in the study published in *The Plant Cell* last year:

The penetrance of the growth phenotype in *dcl4* mutants is directly proportional, not just “correlated”, over at least a 20-fold expression amplitude of DCL2 protein (Nielsen et al. 2024), accurately quantified by targeted mass spectrometry. Mutants heterozygous for *dcl2* knockout alleles have 0.5-fold the amount of DCL2 protein detected in wild type, and the penetrance measured as the odds of having the growth inhibition phenotype in the population decreases proportionately. Similar observations apply to various degrees of DCL2 overexpression (3-fold, 5-fold, 10-fold, and 20-fold) (Fig. 2A, B, and C). By contrast, levels of different DCL2-dependent siRNAs do not show this linear dependence on DCL2 protein levels, as clearly noted in the paper (Nielsen et al. 2024) (Fig. 2D). This observation strongly indicates that the growth restriction phenotype is directly related to DCL2 protein properties. Specifically, it is inconsistent with a silencing effect of its products that involves several intermediary steps, including:

- loading into an AGO protein to produce an active RNA Induced Silencing Complex (RISC),
- the silencing process itself by RISC.

Evidence for importance of the loading step as decisive for the steady state levels of many small RNAs is particularly compelling. It has been observed directly in insect cell culture (Reichholf et al. 2019) and is inferred from many observations in Arabidopsis (Mallory and Vaucheret 2009). These observations also include limited effects on steady state accumulation of several miRNAs even in quite strong loss-of-function mutants in *DCL1* (Liu et al. 2008), clearly indicating the existence of a bottle-neck decisive for small RNA accumulation downstream of the DCL-mediated biogenesis step. These considerations and the results on siRNA levels in Fig. 2D show that the silencing output is not a linear function of DCL concentration, neither in general, nor in the specific case of DCL2 in a *dcl4* mutant background. But in this latter case, the physiological output (penetrance of growth restriction phenotype) is in fact linearly dependent on DCL2 concentration over at least a 20-fold range, all but excluding a silencing effect by DCL2-mediated siRNAs as the cause of growth restriction.

Thus, we are left with two possibilities. Either the DCL2 protein does something completely unrelated to small RNA processing to bring about growth restriction, or the direct catalytic turnover (not the levels of resulting siRNAs), proportional to enzyme concentration in Michaelis–Menten kinetics, is the relevant property that brings about growth restriction. Because we show that growth restriction and basal antiviral defense depend completely on RNaseIII catalytic residues and on residues required for ATP-binding in the helicase domain, our results point strongly to the catalytic turnover as the event that produces the signal to bring about growth restriction through activation of innate immunity (Nielsen et al. 2024).

### Point 3. Use of *dcl2* point mutants isolated by forward genetics

**Figure 3.**
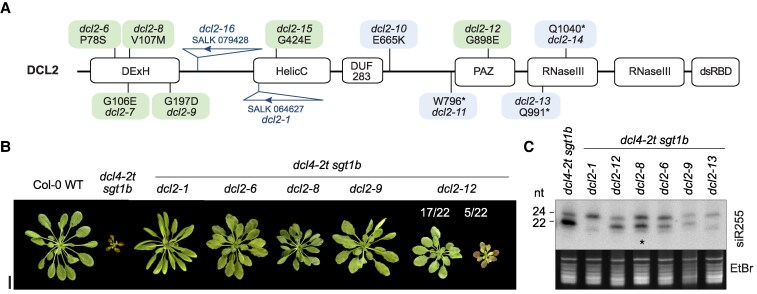
Phenotypes of *dcl2* point mutants that suppress growth defects in *dcl4-2t sgt1b*. **A)** Schematic overview of the position of the T-DNA insertion in the *DCL2* knockout lines *dcl2-1* and *dcl2-16*, and the location of the mutations in the *dcl2* alleles recovered by forward genetic screening for *dcl4-2t sgt1b* suppressors. Green, missense mutations resulting in at least partial Dicer activity; blue, missense or nonsense mutations resulting in complete loss of Dicer activity. Domain designations: DExH and HelicC, helicase domains; DUF283, Dicer dsRNA-binding fold; PAZ, Piwi–Argonaute–Zwille domain; RNase III, RNase III domains; dsRBD, dsRNA-binding domain. **B)** 49-day-old rosettes of the indicated genotypes grown under short-day conditions. Scale bar, 2 cm. **C)** Small RNA blot hybridized to a radiolabeled probe complementary to the trans-acting siRNA siR255. Ethidium bromide (EtBr) staining of the upper part of the gel is shown as loading control. The results shown in this figure were first published as elements of Figures 6 and 7 in our 2024 Plant Cell paper (Nielsen et al. 2024).

Our use of *dcl2* point mutants isolated by forward genetics is criticized by Liu et al., because none of them achieves full decoupling between siRNA production and suppression of growth restriction. We agree and, exactly for that reason, we take care not to over-conclude on the basis of these mutants alone in our report (Nielsen et al. 2024). Nonetheless, as noted above, the results still show that it is possible to find *dcl2* mutants that must have appreciable enzymatic activity in vivo since DCL2-dependent siRNAs produced in wild type are almost as abundant in the mutants, despite the fact that the mutants nearly completely suppress the growth phenotype (Fig. 3). This observation does make models advocating siRNA generation for silencing as the causal role of DCL2 hard pressed to find a convincing explanation. On the other hand, it is readily explained by our model that the catalytic turnover itself (by acting as a signal) underlies the growth phenotypes.

### Point 4. Inactivation of NLRs suppresses growth phenotypes in *dcl4* and *dcl4 sgt1b* mutants

Liu et al. further comment on our evidence presented in the preprint that d*cl4* mutants are characterized by an autoimmune state, and that the growth phenotypes are caused by NLR activation, specifically by the two NLRs L5 and RPP9 (Nielsen et al. 2023). They concede that inactivation of these NLRs does indeed suppress the growth restriction observed in *dcl4* and *dcl4 sgt1b*. Hence, we can all agree that these NLRs are necessary for the growth restriction phenotype, a major step forward. The main argument now becomes the following: How do we know whether silencing of some host factor by DCL2-dependent siRNAs, perhaps *SMXL4/5* or *NIA1/2*, causes NLR activation indirectly? This is a valid concern: NLRs typically detect pathogens indirectly as they respond to changes in those host factors that are targeted by pathogen virulence factors to establish infection (Jones and Dangl 2006). Hence, autoimmune mutants caused by NLR activation through genetic inactivation of virulence factor targets are common, and have indeed caused much confusion in interpretation of phenotypes over the years (Lolle et al. 2017). But Liu et al. only mention our finding that *l5* and *rpp9* mutants suppress growth phenotypes and autoimmune activation in *dcl4* and *dcl4 sgt1b* mutant backgrounds, and fail to mention several other results, pivotal to address this point. A fairer summary of all the relevant results would be the following:

- *dcl4* mutants not only in Arabidopsis but also in tomato and, remarkably, the moss *Physcomitrium patens* exhibit growth defects and a clear defense-related gene expression profile with overrepresentation of the W-box, a cis-regulatory element binding the defense-related WRKY transcription factors, in promoters of differentially expressed genes. Interestingly, in *P. patens* which, similar to other bryophytes, does not encode the *DCL2* clade, this autoimmune state is suppressed by inactivation of the *DCL3* gene (Nielsen et al. 2023). It would already be a remarkable coincidence if silencing of more or less random host genes in both tomato and Arabidopsis would lead to NLR activation. But with the *P. patens* results, this scenario becomes virtually impossible, not least because *P. patens* neither encodes a 22-nt siRNA-producing DCL2 that could initiate excessive feedback amplification, nor does it encode the SMXL4/SMXL5 proteins.
- The growth phenotype of *dcl4* or *dcl4 sgt1b* can be strongly suppressed by inactivation of either one of two nucleotide-binding leucine repeat intracellular immune receptors, *L5* and *RPP9* (Nielsen et al. 2023).
- Inactivation of *RPP9* or *L5* has no effect on infectivity of a positive-strand RNA virus rendered uninfectious on wild type plants by deletion of its anti-RNAi virulence factor. But, crucially, together with inactivation of *dcl4*, knockout of either *RPP9* or *L5* breaks this basal antiviral resistance, in the case of *L5* detectably, if relatively weakly; in the case of *RPP9* with an effect size approaching that of inactivation of *DCL2* itself. Furthermore, viral titers are similar in *dcl4 dcl2* and *dcl4 dcl2 rpp9*, consistent with DCL2 and RPP9 acting in the same antiviral pathway (Nielsen et al. 2023).

This key result provides strong genetic evidence for the relevance of L5 and RPP9 in basal antiviral resistance, a result that one would not expect if their activation in *dcl4* mutants were a fortuitous result of ectopic silencing of random endogenous genes by DCL2-dependent siRNAs. On the other hand, this is precisely what is predicted from our model of DCL2-mediated dsRNA sensing. How that sensing occurs mechanistically, including exactly how RPP9 and L5 are activated dependent on dsRNA processing by DCL2 are now questions of extraordinary significance, and we actively pursue acquisition of knowledge to answer these questions in a satisfactory manner.

#### The variable nature of autoimmune phenotypes

In connection with the introduction of autoimmunity as the cause of DCL2-dependent growth phenotypes, Liu et al. raise three further points that we wish to briefly comment on.

- The first relates to variability of DCL2-dependent growth phenotypes in *dcl4*, *dcl4 xrn4*, and *dcl4 ski2* mutants. Liu et al. are concerned that autoimmunity cannot explain such phenotypic variability, as they write “*… this theory [auto-immunity induced through DCL2 processing of dsRNA] is difficult to explain the various types and severities of growth defects of dcl4 as well as dcl4 xrn4 and dcl4 ski2.*” We are puzzled by this comment, because in fact, many NLR-dependent Arabidopsis autoimmune mutants show phenotypic variability and pronounced dependence on growth conditions (Yu et al. 1998; Brodersen et al. 2002; Zhang et al. 2003; Palma et al. 2010; Lolle et al. 2017). Furthermore, the 5′-3′ exonuclease XRN4 and the DExH helicase SKI2 are major components of distinct cytoplasmic RNA decay systems (Souret et al. 2004; Yu et al. 2016; Lange et al. 2019; Lange and Gagliardi 2021). In single mutants of these genes, many RDR6-dependent siRNAs are produced (Gregory et al. 2008; Branscheid et al. 2015), so it is not hard to imagine strongly increased dsRNA levels, perhaps in distinct cytoplasmic localizations, in *dcl4 xrn4* and *dcl4 ski2* mutants. This could lead to different strengths of NLR activation through DCL2 processing, perhaps even distinct NLRs depending on the subcellular site of processing. Whether such scenarios are true remains unknown at this point, but clearly, the observed phenotypic variability and the enhancement of growth defects in *dcl4* by *ski2* and *xrn4* mutations do not constitute arguments against the model of autoimmunity induced by DCL2-mediated dsRNA processing.
- The second point concerns the timing of the on-set of growth phenotypes, related to the first point of variability. Liu et al. seem to be particularly concerned that we report growth phenotypes of *dcl4 sgt1b* in fairly old (6 to 7 weeks) rosettes. There is no mystery, however. As reported in the papers (Nielsen et al. 2023, 2024), when plants are grown under short days, phenotypes are documented after 6 to 7 weeks of growth. We developed this routine for suppression of growth phenotypes in *dcl4 sgt1b*, because we noticed that the on-set of phenotypes was a bit more homogenous under short-day conditions. In addition, genetic backgrounds with suppressed growth defects remain at the rosette stage at the time where nearly all *dcl4 sgt1b* mutants show the phenotype. Together, these properties mean that short-day grown plants lend themselves better to documentation by photography. Nonetheless, phenotypes are also easily visible under long-day conditions, around 3 to 4 weeks of growth.
- Third, Liu et al. are concerned that each of two distinct NLRs, L5 and RPP9, are required nonredundantly for DCL2-dependent autoimmune activation and suggest that this “would require DCL2 to either activate different R proteins or trigger varied signaling pathways through the same R protein…”. A simple interpretation of the suppression of autoimmunity by knockout of either *RPP9* or *L5* is that they act in series, not in parallel. RPP9 is an NLR of the Toll-Interleukin-1 receptor (TIR) class, L5 is an NLR of the coiled-coil (CC) class. Spectacular recent progress on plant NLR function has shown that activated TIR-NLRs are nucleotide-cleaving enzymes that generate signaling nucleotides whose receptors may act as allosteric activators of downstream signaling CC-NLRs (Chai et al. 2023). Whether RPP9-L5 represents such a signaling system remains unknown at this point. Suffice it to say that a model involving serial action of a TIR-NLR and a CC-NLR should not set off alarm bells given the recent advances on plant innate immune signaling.

### Point 5. Further evidence from inactivation of RNAi factors

By the end of their letter, Liu et al. suggest that stronger evidence could be generated by testing what the effect of mutations inactivating AGO1 and HEN1 on *dcl4 sgt1b* may be. HEN1 methylates the 2′-OH of the 3′-nucleotide of all classes of plant siRNAs and miRNAs (Li et al. 2005; Yu et al. 2005), and silencing is significantly reduced in *hen1* mutants (Park et al. 2002; Boutet et al. 2003) because of accelerated degradation of small RNAs (Li et al. 2005). AGO1 is the key protein component of RISC, the actual effector of several types of small RNA-mediated post-transcriptional gene silencing (Vaucheret et al. 2004; Baumberger and Baulcombe, 2005; Qi et al. 2005). If one imagines siRNA-mediated silencing as a linear pathway, as it is often depicted in textbooks representing the mammalian version of it, this would make sense: DCL4 or DCL2 produces siRNAs. Downstream of DCL processing, siRNAs are methylated by HEN1, then loaded into AGO1 that then silences targets. Hence, with this line of thinking, if inactivation of AGO1 and HEN1 suppresses the DCL2-dependent growth phenotype, it would prove that siRNA-mediated silencing, not DCL2 processing is decisive. Indeed, the Guo laboratory has shown that weak alleles of *ago1* suppress growth phenotypes in *dcl4 ski2* mutants (Zhang et al. 2015), and that a *hen1* loss-of-function mutant suppresses growth phenotypes of *dcl4 xrn4* and *dcl4 ski2* mutants (Wu et al. 2020). With this in mind, Liu et al. conclude that “the genetic evidence presented by Wu et al. (2020) demonstrates that 22-nt siRNAs, but not DCL2 protein per se, cause plant growth defects.” But does it really?

#### A circle cannot be represented as a straight line

The problem is that this view overlooks a fundamental property of siRNA-mediated silencing in plants. The plant RNAi system is not linear. It contains an extremely important positive feedback module mediated by AGO1, siRNA, and the RNA-dependent RNA polymerase RDR6 (Vaucheret, 2006), key to understand the DCL2-dependent growth phenotype, because this phenotype depends fully on RDR6, as demonstrated in work from both the Guo laboratory and from us (Zhang et al. 2015; Nielsen et al. 2024). These components can give rise to synthesis of dsRNA from primary siRNA targets. Hence, tampering with any component in this system not only disrupts silencing per se but also decreases the amount of intracellular dsRNA available for processing by DCL2. Thus, results of genetic experiments relying on inactivation of AGO1 or HEN1 cannot distinguish between the models proposed, because these mutations affect both silencing activity and generation of dsRNA for processing by DCL2. Indeed, *ago1* and *hen1* mutants both came out of a genetic screen for mutants defective in RDR6-dependent transgene silencing. In this system, these mutations lead not only to reduced silencing activity, but to a total collapse of the RDR6-dependent siRNA population, demonstrating the power of the positive feedback loop for dsRNA and siRNA generation (Morel et al. 2002; Boutet et al. 2003).

## Conclusion

While we welcome the debate initiated by Liu et al., we find the arguments listed in their letter against the weight of our evidence for RNAi-independent functions of DCL2 in dsRNA sensing to be mostly weak, in some cases unfounded. Importantly, conclusive evidence for the hypothesis favored by the Guo laboratory that silencing of key endogenous genes, such as *SMXL4/5* and *NIA1/2*, causes the DCL2-dependent phenotypes observed in *dcl4* knockout mutants, has simply not been reported. Thus, this model should be treated as a hypothesis against which there is now considerable evidence. Our view remains firm that RNAi-independent functions of DCL2 in cytoplasmic sensing of double-stranded RNA provide the best, in fact the only, currently available model that adequately explains all available evidence. We stress that this does not mean that siRNAs produced by DCL2 cannot be used in RNAi. We very much support the idea that in local infection contexts where DCL4-RNAi has been defeated, dicing by DCL2 induces NLR activation locally and produces siRNAs that act systemically to immunize uninfected tissues through RNAi. As such, it is not impossible that DCL2-dependent siRNAs in some mutant backgrounds may cause consequential silencing under some conditions. But all we can say for sure at this point is that the overwhelming contribution to DCL2-dependent growth inhibition in *dcl4* mutants involves the NLRs RPP9 and L5 that are also required for basal antiviral resistance.

We close by making clear that we do not question the validity of the genetic results from the Guo laboratory on the rescue of lethality of mutants in *dcl4* and RNA decay factors by inactivation of the *DCL2*, *RDR6*, *SGS3*, and *AGO1* genes (Zhang et al. 2015). Indeed, we are impressed by the comprehensive nature of that genetic investigation and find the results to be an important contribution to the field. This debate is concerned exclusively with the interpretation of the results.

## Data Availability

There are no new data associated with this article.

## References

[koaf163-B1] Andika IB, Maruyama K, Sun L, Kondo H, Tamada T, Suzuki N. Differential contributions of plant Dicer-like proteins to antiviral defences against potato virus X in leaves and roots. Plant J. 2015:81(5):781–793. 10.1111/tpj.12770 25619543

[koaf163-B2] Baumberger N, Baulcombe DC. Arabidopsis ARGONAUTE1 is an RNA Slicer that selectively recruits microRNAs and short interfering RNAs. Proc Natl Acad Sci U S A. 2005:102(33):11928–11933. 10.1073/pnas.0505461102 16081530 PMC1182554

[koaf163-B3] Bouché N, Lauressergues D, Gasciolli V, Vaucheret H. An antagonistic function for Arabidopsis DCL2 in development and a new function for DCL4 in generating viral siRNAs. EMBO J. 2006:25(14):3347–3356. 10.1038/sj.emboj.7601217 16810317 PMC1523179

[koaf163-B4] Boutet S, Vazquez F, Liu J, Béclin C, Fagard M, Gratias A, Morel JB, Crété P, Chen X, Vaucheret H. Arabidopsis HEN1: a genetic link between endogenous miRNA controlling development and siRNA controlling transgene silencing and virus resistance. Curr Biol. 2003:13(10):843–848. 10.1016/S0960-9822(03)00293-8 12747833 PMC5137371

[koaf163-B5] Branscheid A, Marchais A, Schott G, Lange H, Gagliardi D, Andersen SU, Voinnet O, Brodersen P. SKI2 mediates degradation of RISC 5′-cleavage fragments and prevents secondary siRNA production from miRNA targets in Arabidopsis. Nucleic Acids Res. 2015:43(22):10975–10988. 10.1093/nar/gkv1014 26464441 PMC4678812

[koaf163-B6] Brodersen P, Petersen M, Pike HM, Olszak B, Skov S, Odum N, Jørgensen LB, Brown RE, Mundy J. Knockout of Arabidopsis *ACCELERATED-CELL-DEATH11* encoding a sphingosine transfer protein causes activation of programmed cell death and defense. Genes Dev. 2002:16(4):490–502. 10.1101/gad.218202 11850411 PMC155338

[koaf163-B7] Chai J, Song W, Parker JE. New biochemical principles for NLR immunity in plants. Mol Plant Microbe Interact. 2023:36(8):468–475. 10.1094/MPMI-05-23-0073-HH 37697447

[koaf163-B8] Chen H-M, Chen L-T, Patel K, Li Y-H, Baulcombe DC, Wu S-H. 22-nucleotide RNAs trigger secondary siRNA biogenesis in plants. Proc Natl Acad Sci U S A. 2010:107(34):15269–15274. 10.1073/pnas.1001738107 20643946 PMC2930544

[koaf163-B9] Cuperus JT, Carbonell A, Fahlgren N, Garcia-Ruiz H, Burke RT, Takeda A, Sullivan CM, Gilbert SD, Montgomery TA, Carrington JC. Unique functionality of 22-nt miRNAs in triggering RDR6-dependent siRNA biogenesis from target transcripts in Arabidopsis. Nat Struct Mol Biol. 2010:17(8):997–1003. 10.1038/nsmb.1866 20562854 PMC2916640

[koaf163-B10] Deleris A, Gallego-Bartolome J, Bao J, Kasschau KD, Carrington JC, Voinnet O. Hierarchical action and inhibition of plant Dicer-like proteins in antiviral defense. Science. 2006:313(5783):68–71. 10.1126/science.1128214 16741077

[koaf163-B11] Ding SW, Voinnet O. Antiviral immunity directed by small RNAs. Cell. 2007:478(3):413–426. 10.1016/j.cell.2007.07.039 PMC2703654 17693253

[koaf163-B12] Fei Q, Yu Y, Liu L, Zhang Y, Baldrich P, Dai Q, Chen X, Meyers BC. Biogenesis of a 22-nt microRNA in Phaseoleae species by precursor-programmed uridylation. Proc Natl Acad Sci U S A. 2018:115(31):8037–8042. 10.1073/pnas.1807403115 30012624 PMC6077734

[koaf163-B13] Gasciolli V, Mallory AC, Bartel DP, Vaucheret H. Partially redundant functions of Arabidopsis DICER-like enzymes and a role for DCL4 in producing trans-acting siRNAs. Curr Biol. 2005:16(16):1494–1500. 10.1016/j.cub.2005.07.024 16040244

[koaf163-B14] Gregory BD, O’Malley RC, Lister R, Urich MA, Tonti-Filippini J, Chen H, Millar AH, Ecker JR. A link between RNA metabolism and silencing affecting Arabidopsis development. Dev Cell. 2008:14(6):854–866. 10.1016/j.devcel.2008.04.005 18486559

[koaf163-B15] Jones JD, Dangl JL. The plant immune system. Nature. 2006:444(7117):323–329. 10.1038/nature05286 17108957

[koaf163-B16] Lange H, Gagliardi D. Catalytic activities, molecular connections, and biological functions of plant RNA exosome complexes. Plant Cell. 2021:34(3):967–988. 10.1093/plcell/koab310 PMC8894942 34954803

[koaf163-B17] Lange H, Ndecky SYA, Gomez-Diaz C, Pflieger D, Butel N, Zumsteg J, Kuhn L, Piermaria C, Chicher J, Christie M, et al RST1 and RIPR connect the cytosolic RNA exosome to the Ski complex in Arabidopsis. Nat Commun. 2019:10(1):3871. 10.1038/s41467-019-11807-4 31455787 PMC6711988

[koaf163-B18] Li J, Yang Z, Yu B, Liu J, Chen X. Methylation protects miRNAs and siRNAs from a 3′-end uridylation activity in Arabidopsis. Curr Biol. 2005:15(16):1501–1507. 10.1016/j.cub.2005.07.029 16111943 PMC5127709

[koaf163-B19] Liu Q, Yao X, Pi L, Wang H, Cui X, Huang H. The ARGONAUTE10 gene modulates shoot apical meristem maintenance and leaf polarity establishment by repressing miR165/166 in Arabidopsis. Plant J. 2008:58(1):27–40. 10.1111/j.1365-313X.2008.03757.x 19054365

[koaf163-B20] Lolle S, Greeff C, Petersen K, Roux M, Jensen MK, Bressendorff S, Rodriguez E, Sømark K, Mundy J, Petersen M. Matching NLR immune receptors to autoimmunity in *camta3* mutants using antimorphic NLR alleles. Cell Host Microbe. 2017:21(4):518–529.e514. 10.1016/j.chom.2017.03.005 28407487

[koaf163-B21] Mallory AC, Vaucheret H. ARGONAUTE 1 homeostasis invokes the coordinate action of the microRNA and siRNA pathways. EMBO Rep. 2009:10(5):521–526. 10.1038/embor.2009.32 19343050 PMC2680873

[koaf163-B22] Morel J-B, Gordon C, Mourrain P, Béclin C, Boutet S, Feuerbach F, Proux F, Vaucheret H. Fertile hypomorphic *ARGONAUTE* (*ago1*) mutants impaired in post-transcriptional gene silencing and virus resistance. Plant Cell. 2002:14(3):629–639. 10.1105/tpc.010358 11910010 PMC150585

[koaf163-B23] Nielsen CPS, Arribas-Hernández L, Han L, Reichel M, Woessmann J, Daucke R, Bressendorff S, López-Márquez D, Andersen SU, Pumplin N, et al Evidence for an RNAi-independent role of Arabidopsis DICER-LIKE2 in growth inhibition and basal antiviral resistance. Plant Cell. 2024:36(6):2289–2309. 10.1093/plcell/koae067 38466226 PMC11132882

[koaf163-B24] Nielsen CPS, Han L, Arribas-Hernández L, Karelina D, Petersen M, Brodersen P. Sensing of viral RNA in plants via a DICER-LIKE Ribonuclease. bioRxiv 523395. 10.1101/2023.01.10.523395, 11 January 2023, preprint: not peer reviewed.

[koaf163-B25] Palma K, Thorgrimsen S, Malinovsky FG, Fiil BK, Nielsen HB, Brodersen P, Hofius D, Petersen M, Mundy J. Autoimmunity in Arabidopsis acd11 is mediated by epigenetic regulation of an immune receptor. PLoS Pathog. 2010:6(10):e1001137. 10.1371/journal.ppat.1001137 20949080 PMC2951382

[koaf163-B26] Parent JS, Bouteiller N, Elmayan T, Vaucheret H. Respective contributions of Arabidopsis DCL2 and DCL4 to RNA silencing. Plant J. 2015:81(2):223–232. 10.1111/tpj.12720 25376953

[koaf163-B27] Park W, Li J, Song R, Messing J, Chen X. CARPEL FACTORY, a Dicer homolog, and HEN1, a novel protein, act in microRNA metabolism in *Arabidopsis thaliana*. Curr Biol. 2002:12(17):1484–1495. 10.1016/S0960-9822(02)01017-5 12225663 PMC5137372

[koaf163-B28] Qi Y, Denli AM, Hannon GJ. Biochemical specialization within Arabidopsis RNA silencing pathways. Mol Cell. 2005:19(3):421–428. 10.1016/j.molcel.2005.06.014 16061187

[koaf163-B29] Reichholf B, Herzog VA, Fasching N, Manzenreither RA, Sowemimo I, Ameres SL. Time-resolved small RNA sequencing unravels the molecular principles of MicroRNA homeostasis. Mol Cell. 2019:75(4):756–768.e757. 10.1016/j.molcel.2019.06.018 31350118 PMC6713562

[koaf163-B30] Sakurai Y, Baeg K, Lam AYW, Shoji K, Tomari Y, Iwakawa H-O. Cell-free reconstitution reveals the molecular mechanisms for the initiation of secondary siRNA biogenesis in plants. Proc Natl Acad Sci U S A. 2021:118(31):e2102889118. 10.1073/pnas.2102889118 34330830 PMC8346886

[koaf163-B31] Souret FF, Kastenmayer JP, Green PJ. AtXRN4 degrades mRNA in Arabidopsis and its substrates include selected miRNA targets. Mol Cell. 2004:15(2):173–183. 10.1016/j.molcel.2004.06.006 15260969

[koaf163-B32] Vaucheret H . Post-transcriptional small RNA pathways in plants: mechanisms and regulations. Genes Dev. 2006:20(7):759–771. 10.1101/gad.1410506 16600909

[koaf163-B33] Vaucheret H, Vazquez F, Crété P, Bartel DP. The action of ARGONAUTE1 in the miRNA pathway and its regulation by the miRNA pathway are crucial for plant development. Genes Dev. 2004:18(10):1187–1197. 10.1101/gad.1201404 15131082 PMC415643

[koaf163-B34] Wang XB, Jovel J, Udomporn P, Wang Y, Wu Q, Li WX, Gasciolli V, Vaucheret H, Ding SW. The 21-nucleotide, but not 22-nucleotide, viral secondary small interfering RNAs direct potent antiviral defense by two cooperative argonautes in *Arabidopsis thaliana*. Plant Cell. 2011:23(4):1625–1638. 10.1105/tpc.110.082305 21467580 PMC3101545

[koaf163-B35] Wu H, Li B, Iwakawa H-O, Pan Y, Tang X, Ling-hu Q, Liu Y, Sheng S, Feng L, Zhang H, et al Plant 22-nt siRNAs mediate translational repression and stress adaptation. Nature. 2020:581(7806):89–93. 10.1038/s41586-020-2231-y 32376953

[koaf163-B36] Wu Y-Y, Hou B-H, Lee W-C, Lu S-H, Yang C-J, Vaucheret H, Chen H-M. DCL2- and RDR6-dependent transitive silencing of SMXL4 and SMXL5 in Arabidopsis dcl4 mutants causes defective phloem transport and carbohydrate over-accumulation. Plant J. 2017:90(6):1064–1078. 10.1111/tpj.13528 28267232

[koaf163-B37] Yoshikawa M, Han Y-W, Fujii H, Aizawa S, Nishino T, Ishikawa M. Cooperative recruitment of RDR6 by SGS3 and SDE5 during small interfering RNA amplification in *Arabidopsis*. Proc Natl Acad Sci U S A. 2021:118(34):e2102885118. 10.1073/pnas.2102885118 34408020 PMC8403909

[koaf163-B38] Yoshikawa M, Iki T, Tsutsui Y, Miyashita K, Poethig RS, Habu Y, Ishikawa M. 3′ fragment of miR173-programmed RISC-cleaved RNA is protected from degradation in a complex with RISC and SGS3. Proc Natl Acad Sci U S A. 2013:110(10):4117–4122. 10.1073/pnas.1217050110 23417299 PMC3593843

[koaf163-B39] Yu B, Yang Z, Li J, Minakhina S, Yang M, Padgett RW, Steward R, Chen X. Methylation as a crucial step in plant microRNA biogenesis. Science. 2005:307(5711):932–935. 10.1126/science.1107130 15705854 PMC5137370

[koaf163-B40] Yu IC, Parker J, Bent AF. Gene-for-gene disease resistance without the hypersensitive response in *Arabidopsis dnd1* mutant. Proc Natl Acad Sci U S A. 1998:95(13):7819–7824. 10.1073/pnas.95.13.7819 9636234 PMC22769

[koaf163-B41] Yu X, Willmann MR, Anderson SJ, Gregory BD. Genome-wide mapping of uncapped and cleaved transcripts reveals a role for the nuclear mRNA cap-binding complex in cotranslational RNA decay in Arabidopsis. Plant Cell. 2016:28(10):2385–2397. 10.1105/tpc.16.00456 27758893 PMC5134982

[koaf163-B42] Zhang X, Zhu Y, Liu X, Hong X, Xu Y, Zhu P, Shen Y, Wu H, Ji Y, Wen X, et al Suppression of endogenous gene silencing by bidirectional cytoplasmic RNA decay in Arabidopsis. Science. 2015:348(6230):120–123. 10.1126/science.aaa2618 25838384

[koaf163-B43] Zhang Y, Goritschnig S, Dong X, Li X. A gain-of-function mutation in a plant disease resistance gene leads to constitutive activation of downstream signal transduction pathways in suppressor of npr1–1, constitutive 1. Plant Cell. 2003:15(11):2636–2646. 10.1105/tpc.015842 14576290 PMC280567

